# Low dietary diversity and its influencing factors among a San group in Namibia

**DOI:** 10.1186/s13104-019-4408-8

**Published:** 2019-06-28

**Authors:** Anita Heim, Attila Paksi

**Affiliations:** 1 Department of Agricultural Sciences, University of Helsinki, PL 27 (Latokartanonkaari 5), Room 222, 00014 Helsinki, Finland; 2 Department of Political and Economic Studies, University of Helsinki, Unioninkatu 37, P.O. Box 54, 00014 Helsinki, Finland

**Keywords:** San, Dietary diversity, Namibia, Socioeconomic factors, Diet quality

## Abstract

**Objective:**

Although the San in Namibia have been targeted by intensive development efforts, there is little knowledge available about San diet quality and nutritional status. The objective of this study is therefore to estimate and quantify the dietary diversity of a San group, and to investigate how socioeconomic characteristics affect dietary diversity. The dietary data (n = 200) for this cross-sectional study were collected as a part of a larger doctoral research investigating food environment, food choices, and dietary changes of the Khwe San in Bwabwata National Park East.

**Results:**

The mean dietary diversity score (DDS) of the participants was 2.44 out of 10, with only two people having a DDS of 5. 87.5% of participants consumed only from 2 or 3 different food groups, mainly grains/roots and dark green leafy vegetables. DDS significantly correlated only with the level of education and with age. Due to their collinearity, the group with no education had the lowest DDS, but also belonged to the oldest age group. The overall dietary diversity of the Khwe is extremely low, indicating severe nutritional inadequacy. The small differences in DDS among the socioeconomic groups indicate the importance of other determining factors, such as cultural and food environmental characteristics.

## Introduction

In 2001, the San people—a generic term for several Southern African Indigenous ethnic groups—were regarded as a severe food insecure group in Namibia [1]. The different San groups share similar challenges: all have experienced deprivation of land and natural resources, have limited ability to practise their traditional livelihood based on hunting and gathering, are subject to regular discrimination, have low social status, and have only a short history of farming practices [2]. Since 2005 Namibia has been implementing the San Development Programme (SDP), with a substantial and annually increasing budget [2]. One would expect that the food and nutrition security of the San would have improved over the past two decades, however, there are no quantitative studies that have reported on the nutritional status and characteristics of the diets of any San groups since the early 2000s.

It must be noted, that a food and nutrition security monitoring system (FNSMS) operates in Namibia [3, 4], but it does not offer information on specific ethnic groups; data collection by ethnicity in national surveys is prohibited by law [5], as ethnic distinction is regarded as a legacy of apartheid. Yet, the FNSMS annual reports highlight the extremely poor diet quality and micronutrient intake in the most food insecure regions [4].

Food and nutrition security is a multidimensional concept, with one of its dimensional measures being the quality of diets [6]. Dietary diversity score (DDS) is a widely used numerical indicator of diet quality [7–9]. It is imperative to assess dietary diversity within the most food insecure populations, to provide feedback and information to development efforts.

The Khwe people—one of the San groups residing in the Zambezi and Kavango East Regions—are one of the most marginalized communities in Namibia. They have been a target of the SDP from its beginning. To improve their socioeconomic status, young people have received support to enter education programs, elders have been hired as community game guards, and ministries have been encouraged to employ Khwe community members [2]. Furthermore, the SDP aimed to diversify livelihood options through community gardening, community bakery, farming training and input, provision of food aid, and the donation of small livestock. These projects are also expected to increase local food availability, but their impacts on actual food intake have been overlooked. To date, no studies of the contemporary diet quality or nutritional status of the Khwe have been undertaken.

The present study forms a part of a larger doctoral research investigating food environment, food choices, and dietary changes of the Khwe San in Bwabwata National Park (BNP) East. The initial aim of the dietary data collection was to establish baseline dietary information on the adult population. However, given the large research gap in dietary assessments among San communities, this paper set out to (i) estimate and quantify the dietary diversity of the Khwe, and (ii) investigate how socioeconomic characteristics affect their dietary diversity.

## Main text

### Method

#### Study population

The former hunter-gatherer Khwe people are among the few Indigenous groups in Africa who are still living on their ancestral land; however, in contrast to their previous nomadic lifestyle, they have been pressured to reside in permanent settlements since the 1970s [10]. Today, their land is within a State designated National Park, and they are prohibited from hunting and are severely restricted in wild food collection. Only 16.9% of the working-age population is currently employed [11]. Today, the livelihood of the approximately 1600 individuals who live in the East BNP is supported by irregular food aid deliveries and monthly social grants from the government. Through the countrywide community-based natural resource management (CBNRM) initiative, the Khwe community receives some economic and food benefits from trophy hunting concessions, e.g. game meat twice a year. Due to the remoteness of the villages in the study area (the nearest market town is 160 km away), the community members have access to a mobile shop once a month, while the few local village shops sell only non-perishable food items and alcohol.

#### Data collection

To describe the present diet we used single administered qualitative 24-h dietary recalls with 200 participants, selected randomly from a list of all adults (752) in the study area based on a previously administered census of the total population. The sample size was pre-calculated with a confidence level of 90% and a margin of error of 5%. The recalls were conducted both in the rainy season (n = 91) and the dry seasons (n = 109) between April 2017 and April 2018. Data on socioeconomic status (SES) was collected, while valuable information about dietary practices was obtained through participant observation.

In our study we adhered the ethical guidelines of the International Society of Ethnobiology [12]. Permission to undertake the study was granted by the community leaders, with participation of all individuals being entirely voluntary. Participants were asked for their written free, prior, and informed consent.

### Data analysis

DDS was derived from the 24-h dietary recalls and represents the number of different food groups consumed over a certain reference period [13]. Food groups (see Table 2) were defined according to FAO guidelines [14]. A minimum of 1 tablespoon of any food items consumed was assigned to a defined food group for each score, and the number of different food groups consumed was summed to construct the DDS for each 24-h recall. The maximum score was 10. The higher the score, the better the quality of the individual’s diet. The association of SES variables and DDS was statistically tested in SPSS version 22.0 (IBM Corp., Chicago, IL, USA), using Spearman correlation and Mann–Whitney U tests. Confounding factors were tested with analysis of covariance. A *P* value below 0.05 was considered to be significant.

### Results

In total, 200 adults participated in the dietary recall survey: 111 women—82 of them reproductive-aged (18–49 years)—and 89 men, with an average age of 39 years. Of these, only 22 participants were employed during the data collection period, with the majority of respondents living in low-income households. Seventy-eight participants had access to a small cultivation plot—usually less than 1 hectare—and 91 respondents had a few chickens or goats (Table 1).

**Table 1:** Characteristics of the total sample (n = 200) and DDS by gender in BNP East, Namibia

	Total			Women (n = 111)			Men (n = 89)			P^#^
	n	DDS^a^	SD	n	DDS^a^	SD	N	DDS^a^	SD	
Age										
18–49	144	2.5	0.72	82	2.46	0.757	62	2.55	0.67	0.377
50+	56	2.27	0.78	29	2.38	0.82	27	2.15	0.662	0.278
Education level										
No education	64	2.20*	0.74	39	2.28	0.826	25	2.08	0.572	0.356
Low (Grade 1–7)	78	2.55	0.73	50	2.5	0.735	28	2.64	0.731	0.344
High (Grade 8+)	58	2.53	0.68	22	2.59	0.734	36	2.5	0.655	0.787
Employment										
Employed	22	2.18	0.79	8	2.13	0.991	14	2.21	0.699	0.856
Unemployed	178	2.47	0.72	103	2.47	0.752	75	2.47	0.684	0.630
Income^b^										
Very low (US$ 0–1.9/day)	148	2.43	0.73	82	2.48	0.789	66	2.38	0.651	0.681
Low (US$ 1.9–3.8/day)	35	2.37	0.81	21	2.29	0.784	14	2.5	0.855	0.523
Medium (US$ 3.8/day+)	17	2.59	0.62	8	2.5	0.535	9	2.67	0.707	0.666
Agricultural field^c^										
Yes	78	2.35	0.75	40	2.25	0.742	38	2.45	0.76	0.238
No	122	2.49	0.72	71	2.55	0771	51	2.41	0.638	0.431
Livestock^d^										
Yes	91	2.45	0.75	50	2.42	0.785	41	2.49	0.711	0.499
No	109	2.42	0.73	61	2.46	0.765	48	2.38	0.672	0.619

^#^*P* values for the comparison of men and women, calculated using Mann–Whitney U tests. Significant if P < 0.05

*Post-hoc test reveals that the mean DDS of the ‘no education’ subgroup differs significantly from the other two subgroups in the total sample

^a^Data is presented as means of DDS

^b^Participants were classified into three groups based on monthly household income (very low, low, and medium) using the purchasing power parity equivalent of the World Bank’s poverty ratio of US$1.9 a day per individual

^c^Access to agricultural fields

^d^Access to livestock: goats and chickens

**Fig. 1:**
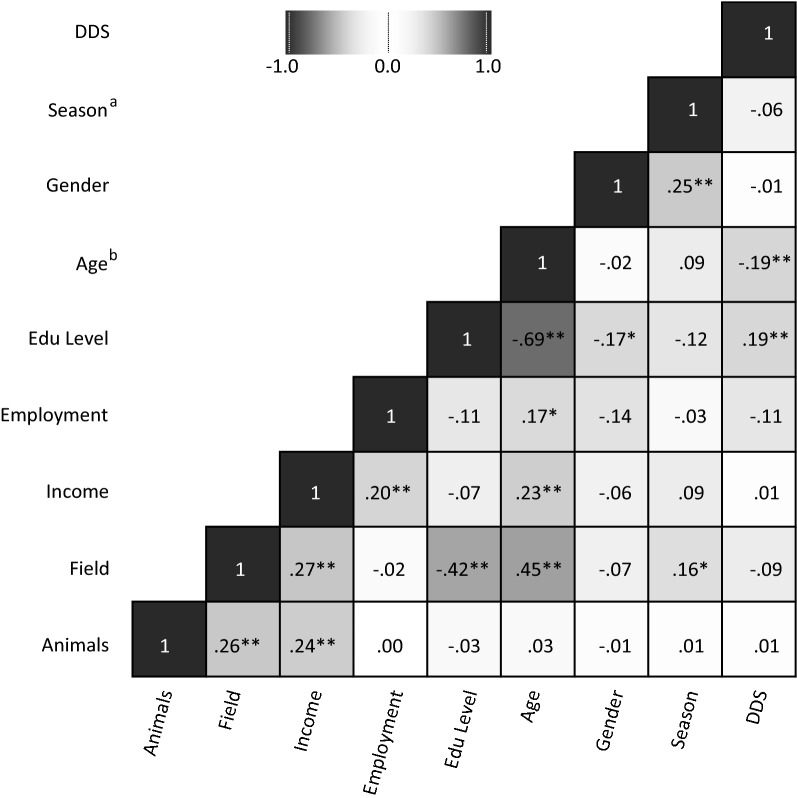
Correlation matrix of DDS and SES variables. ^a^Dichotomous variable: rainy season; dry seasons. ^b^Used as continuous variable. **Correlation is significant at the 0.01 level (two-tailed).*Correlation is significant at the 0.05 level (two-tailed)

DDS ranged from 1 to 5 (of a total score of 10), with an overall mean of 2.44 ± 0.73, with only two cases of individuals consuming foods from 5 different food groups. No significant differences were found when comparing mean DDSs between genders across different SES levels (Table 1). Mean DDS in the rainy season was 2.38 ± 0.79, while in the dry seasons 2.48 ± 0.69. Significant correlation was found between DDS and age and between DDS and educational levels; none of the other SES factors correlated with DDS (Fig. 1). However, multicollinearity was found among several other explanatory variables (e.g.: age-field, age-income, season-gender, see Fig. 1).

The mean DDS across the three different education levels were compared and post hoc test revealed that the group that had no education had significantly lower DDS than the others (Table 1). However, there was a strong correlation between age and education level (r_s_ = − 0.69; Fig. 1), which was also confirmed when including age as a confounding factor into the analysis of covariances. In fact, 81% of the ‘no education’ group were above 50 years old.

Food items from 8 of 10 food groups were consumed; *eggs* and *dairy* were entirely missing from the participants’ diet (Table 2). The two most frequently consumed food groups were *grains/roots*, dominated by maize meal (n = 176, 88%), and *dark green leafy vegetables*, consisting of a variety of 16 wild plants and other easily accessible greens around the village. Meat consumption (n = 55, 27.5%) largely consisted of processed tinned products, while only a few ate vegetables or fruits, representing an overall low level of micro-nutrient intake.

**Table 2:** Food items reportedly consumed by number of individuals (n) in the past 24 h, per food group [14]

Food group	Food items	n
1. Grains/roots	Maize meal	176
	Rice	42
	Bread	11
	Potato	4
	Pasta	3
	Biscuits	1
	Millet	1
	Sorghum	1
	Dinga (*Dioscorea asteriscus*)*	1
	/’iya (*Vigna vexillata*)*	1
2. Pulses	Canned beans	8
	Groundnuts	2
	Dry beans	1
3. Seeds and nuts	Tceu (*Guibourtia coleosperma*)*	12
	/qom (*Schinziophyton rautanenii*)*	5
4. Dairy	–	0
5. Meat/fish	Canned fish	28
	Game meat	22
	Canned processed meat	6
	Chicken	4
	Goat	1
6. Eggs	–	0
7. Dark green leafy vegetables	16 variety of wild greens	76
	Matete (*Hibiscus sabdarifa*)	73
	Cassava leaves	19
	Pumpkin leaves	11
	Derere (*Corchorus tridens*)	10
8. Other vitamin A rich fruits and vegetables	Ce (*Grewia flava*)*	7
	‡ori (*Ximenia americana*)*	6
	Pumpkin	1
9. Other fruits	‡ûmbé (*Dialium engleranum*)*	18
10. Other vegetables	Deu (*Termitomyces* sp.)*	9
	Fresh maize	8
	Tsamma melon (*Citrullus lanatus*)*	3
	Cabbage	3
	Canned vegetables	3
	Onion	2
	Tomato	1

*Local Khwedam names for food gathered from the forest

### Discussion

In this paper, we described the dietary diversity of the Khwe San and examined how SES influence DDS, in order to explore what factors are influencing current diet quality. Of the 200 participants, only two women reported having consumed foods from up to 5 food-groups, which is a threshold for minimum adequate diets for reproductive-aged women (DDS less than 5 is considered under-nourished [14]). The average DDS was below 2.5 across all adult respondents. Given the importance of DDS in determining nutritional status [7, 13], this extremely low dietary diversity suggests high vulnerability and severe nutritional inadequacy in the researched population.

The low DDS derives from the fact that 87.5% of the participants consumed food items from only 2 or 3 different food groups, the most frequently eaten food type being maize meal (Table 2). The Khwe have become habituated to waiting for the food aid deliveries as their primary food source, which mainly consists of maize meal supplemented with few cans of fish, beans, and cooking oil. Green leafy vegetables were frequently consumed in the rainy season due to their easy access, although based on our observations these were often overcooked, meaning most nutrients had leached out before being eaten. Moreover, the sampled Khwe population consumed a very low quantity of seeds, vegetables, and fruits (Table 2). The difference in DDS between the rainy season and the dry seasons was insignificant. Seasons are distinguished according to the distribution of rain, temperature, and some cultural activities. In the recent past though these have changed considerably, and our informants regarded all seasons to be very difficult to obtain enough and diverse food. For example, while in the rainy season the leafy vegetables are dominating their diet, Khwe have no access to meat- as their main source, the trophy hunting meat distribution is only available in the dry seasons. It is important to note that, since 2017, a strict regulation has prohibited the Khwe from entering their primary wild food harvesting areas. These wild plants constituted their main source of vitamins and minerals [15] and were crucial to supplement their otherwise primarily maize meal-based diet. The contemporary Khwe diets were low in non-processed animal flesh foods and totally lacked dairy products and eggs, all of which are considered vital for healthy body development, muscle growth, and nervous system function, and are recommended to be consumed on a regular basis [16].

A number of studies have shown a positive association between level of education and dietary diversity [17–19]. In addition, gender, age, employment status or income were also linked to diet diversity [20]. In this study, the only SES classes that revealed significant correlations were education level and age (Fig. 1). The Khwe participants who obtained any level of education had significantly higher DDSs than those with no education (Table 1). However, most individuals with no education are elderly (50+ years) who also consumed a less diverse diet, than the younger respondents. The strong collinearity between age and education levels indicate that the lower DDS could be a consequence of old age, and not exclusively the result of not obtaining formal education. We initially assumed, that individuals with employment also have higher education; however we found no correlation between these variables. In fact, among the employed respondents several were elderly who worked as community game guards or as trackers for the trophy hunters, jobs that do not require formal education. Livestock owners did not have better diets as livestock are regarded to be a safety net, to sell in times of emergency, rather than a regular source of food [21].

Most of our respondents classify in the low economic status group (Table 1), and the small fraction of people with higher economic status reportedly are expected to look after a large number of relatives. Indeed, similarly to other San groups, the Khwe use their resources and assets in a culturally distinctive manner, which cardinally involves sharing [2]. Individuals with some income or assets tend to share their purchased foods and occasional small agricultural harvest with their relatives and neighbours. Therefore, at the end of the day, the people who are economically better off do not necessarily consume a higher diversity of foods. These examples may partially explain why higher SES does not translate into a substantially better diet quality, and highlights the need to explore other influencing factors of diet, such as cultural and food environmental characteristics.

Indigenous communities undergoing rapid changes in their dietary practices are strongly influenced by the changing characteristics of the local food environment [22]. For the Khwe, the food available in local shops consists mainly of sugar-based foods and beverages, canned meat, rice, noodles, and alcohol, all at high prices. Several of the SDPs have been aimed at raising income, and thereby presumably increasing food accessibility; yet, even for individuals with income, regular access to sufficiently nutritious food remains severely limited. Among indigenous people, nutritious food comes from the traditional diet, supplied by the surrounding environment; however, when more purchased foods are incorporated, diet quality commonly decreases [23–26]. To date, the government has made attempts to develop the community through various SDP projects but these have not translated into dietary diversity high enough for a minimum adequate diet [14]. It seems that acknowledgement and support of traditional food systems must be included into any development strategies assisting the San in order to improve dietary outcomes.

## Limitations

For the aims of the original larger study, the 24-h dietary recall was only single administered, hence not designed to capture day to day or seasonal variation in diet. The relatively small sample size may contribute to the absence of any statistical association between SES and DDS, however our observations and interviews over a period of 15 months reveal a high level of uniformity in the local diet. Our sample includes reproductive aged women as well as men and elders. These variations across age and gender may affect the generalization and the comparison of the results.

## Data Availability

The data analysed during the current study are available from the corresponding author on reasonable request.

## Abbreviations

SDP: San Development Programme
FNSMS: Food and Nutrition Security Monitoring System
BNP: Bwabwata National Park
CBNRM: community-based natural resource management
SES: socioeconomic status
SPSS: Statistical Package for Social Science
FAO: Food and Agriculture Organization of the United Nations
DDS: dietary diversity score
